# Modulation of SRSF2 expression reverses the exhaustion of TILs via the epigenetic regulation of immune checkpoint molecules

**DOI:** 10.1007/s00018-019-03362-4

**Published:** 2019-12-14

**Authors:** Ziqiang Wang, Kun Li, Wei Chen, Xiaoxia Wang, Yikun Huang, Weiming Wang, Wanjun Wu, Zhiming Cai, Weiren Huang

**Affiliations:** 1 Department of Urology, Shenzhen Second People’s Hospital, The First Affiliated Hospital of Shenzhen University, International Cancer Center, Shenzhen University School of Medicine, Shenzhen, 518039 China; 2 Guangdong Key Laboratory of Systems Biology and Synthetic Biology for Urogenital Tumors, Shenzhen, 518035 China; 3 Department of Nuclear Medicine, Shandong Provincial Qianfoshan Hospital, The First Hospital Affiliated with Shandong First Medical University, Jinan, 250014 China

**Keywords:** T-cell exhaustion, Immune checkpoint, SRSF2, Epigenetic regulation, Histone modification

## Abstract

The elevated expression of immune checkpoints by the tumor microenvironment is associated with poor prognosis in several cancers due to the exhaustion of tumor-infiltrating lymphocytes (TILs), and the effective suppression of the expression of these genes is key to reversing the exhaustion of TILs. Herein, we determined that serine/arginine-rich splicing factor 2 (SRSF2) is a target for blocking the tumor microenvironment-associated immunosuppressive effects. We found that the expression of SRSF2 was increased in exhausted T cells and that SRSF2 was involved in multiple immune checkpoint molecules mediating TILs’ exhaustion. Furthermore, SRSF2 was revealed to regulate the transcription of these immune checkpoint genes by associating with an acyl-transferases P300/CBP complex and altering the H3K27Ac level near these genes, thereafter influencing the recruitment of signal transducer and activator of transcription 3 (STAT3) to these gene promoters. Collectively, our data indicated that SRSF2 functions as a modulator of the anti-tumor response of T cells and may be a therapeutic target for reversing the exhaustion of TILs.

**Electronic supplementary material:**

The online version of this article (10.1007/s00018-019-03362-4) contains supplementary material, which is available to authorized users.

## Introduction

Renal cell carcinoma (RCC) is the most common type of kidney cancer and accounts for 90–95% of all kidney cancer diagnosis and 3% of adult malignancies [1]. While there are multiple conventional treatments available for RCC including surgery, chemotherapy, and radiotherapy, there is a subset of RCC patients (30%) that exhibit metastases and are unresponsive to chemotherapy and radiotherapy [2]. It is known that both innate and adaptive immune cells with RCC specificity develop naturally in most patients, and lymphocytes are recruited to the tumor to secrete cytokines [3, 4]. However, higher numbers of T cells in RCC tissue correlates with a poorer prognosis as there are limitations on immune cells and the capacity with which they exert their effector functions [5, 6]. This suggests that for some unknown reasons, RCC T cells are unable to produce an immune response against the tumor cells and control tumor growth. Recent studies have suggested that the immunosuppressive effects of the tumor microenvironment may be the cause of this T-cell failure in RCC tissue.

In the tumor microenvironment, there is an ongoing balance between the cytotoxic T lymphocytes (CTLs) that work to eliminate tumor cells through the secretion of cytokines including IFN-γ and IL-2, and the tumor cells themselves that can negatively regulate CTLs through the association with other immunosuppressive cells like myeloid-derived suppressor cells (MDSCs), tumor-associated macrophages (TAMs), and regulatory T cells (Tregs) [7]. These immunosuppressive cells can secrete cytokines like IL-6, IL-10, TGF-β, and VEGF [8, 9] that can have a direct inhibitory effect on the activation of CTLs by stimulating expression of immune checkpoint molecules that represent cell exhaustion on T cells [10]. There are many immune checkpoint molecules including programmed cell death 1 (PD-1), B- and T-lymphocyte attenuator (BTLA), T-cell immunoglobulin and mucin-domain containing-3 (TIM-3), lymphocyte activation gene 3 (LAG-3), cytotoxic T-lymphocyte antigen-4 (CTLA-4), T-cell immunoreceptor with immunoglobulin, and immunoreceptor tyrosine-based inhibitory motif domains (TIGIT) and CD160. These immune checkpoint molecules contribute to CTLs dysfunction in cytokine secretion, proliferation, tumor cell cytotoxicity, and effective memory cell generation. Therefore, understanding how to directly downregulate different immune checkpoint molecules expression represents a key issue toward reversing CTLs’ dysfunction.

There is a growing body of evidence confirming the involvement of epigenetic regulation in T-cell dysfunction. Research from the Dana Farber Cancer Institute and Harvard-MIT Broad Institute found that BAF 180, a protein localized to the chromatin remodeling complex SWI/SNF, regulates the expression of multiple immune checkpoint molecules, thereby mediating T-cell function and T-cell-mediated anti-tumor immune responses [11, 12]. In addition, histone deacetylase inhibitors also regulate the anti-tumor immune response of tumor-infiltrating CD4 + T cells [13]. These studies reveal that epigenetic mechanisms play an important role in regulating tumor-infiltrating lymphocytes (TILs) function. In this study, we look for epigenetic mechanisms of immune checkpoint molecules that mediate T-cell processes and find that serine/arginine-rich splicing factor 2 (SRSF2) is required for regulating T-cell function.

SRSF2 is an important component of nuclear structure speckle, with many reported functions including genomic stability maintenance, pre-mRNA splicing, and nuclear output regulation and aiding in mRNA translation [14–18]. Through the regulation of cancer-associated splice variants, SRSF2 have been implicated in many types of cancer, including lung carcinoma [19], hepatocellular carcinoma [20], and RCC [21]. Additionally, SRSF2 has been associated with a variety of immune disorders including systemic lupus erythematosus (SLE) [22], leukemia [23], and human immunodeficiency virus infection [24]. Furthermore, SRSF2 is known to regulate gene expression of many T-cell molecules, such as CD45 [25], CD44 [26], and costimulatory signal molecule B7-H3 [27]. Previously, we found that SRSF2 directly binds to genomic DNA to regulate the gene’s transcriptional activity [28], suggesting that SRSF2 may regulate T-cell functions through a similar epigenetic regulation mechanism.

In this study, we found that tumor cells isolated from RCC tissue did not trigger the immune response of TILs and downregulation of SRSF2 in these TILs could significantly improve their immune response against tumor cells. Upon further analysis, we found that RCC TILs overexpressed multiple immune checkpoint molecules, and that inhibition of SRSF2 in TILs led to the downregulation of these immune checkpoint molecules. We found that SRSF2 regulates the transcriptional activities of these genes by altering the nearby H3K27 acetylation via the association with acetyl-transferases P300/CBP complex and signal transducer and activator of transcription 3 (STAT3). All these data suggest that SRSF2 may be a therapeutic target to block tumor-associated suppressive pathways.

## Results

### SRSF2 modulates the immune response against autologous tumor cells in TILs

**Fig. 1:**
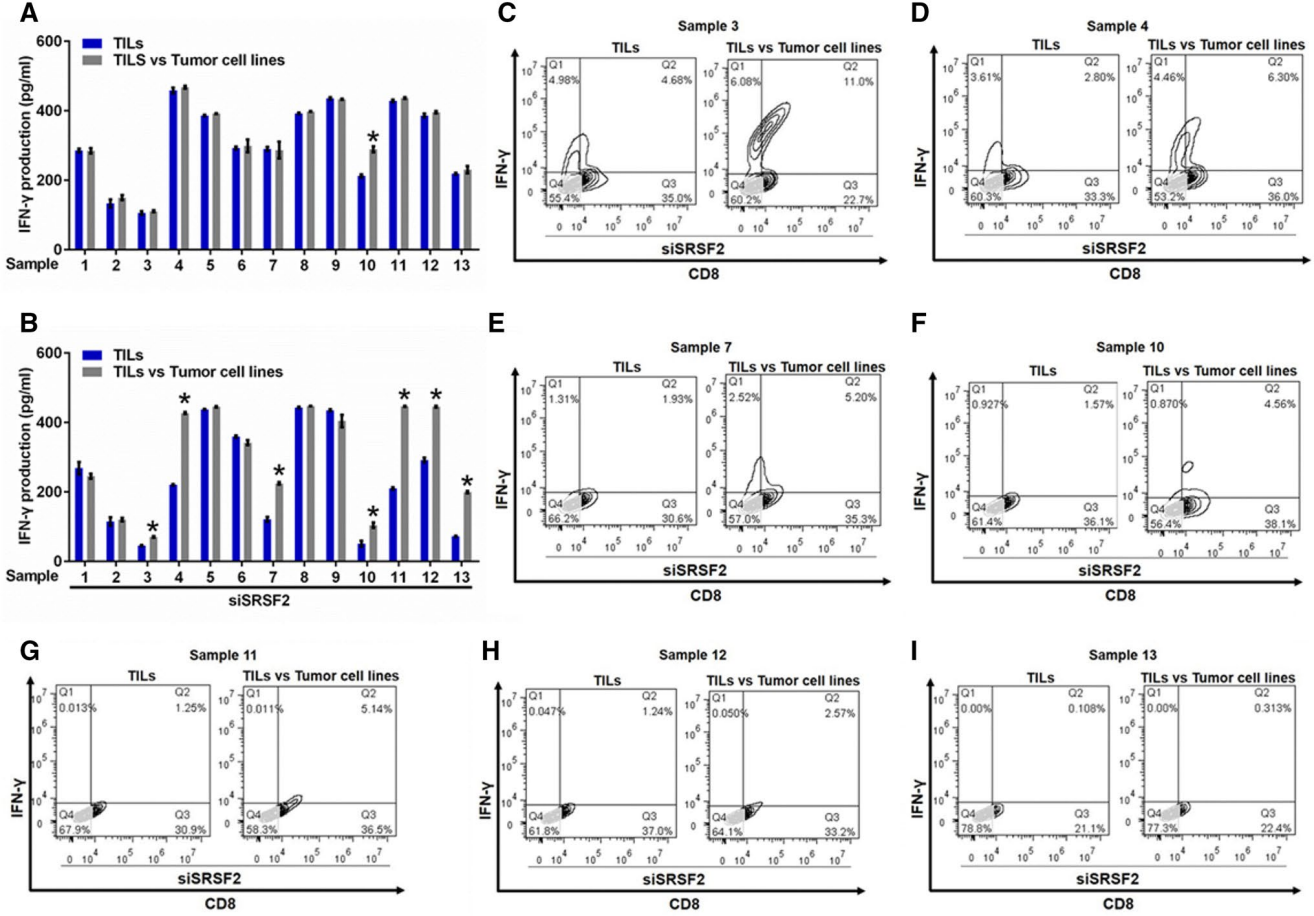
Inhibition of SRSF2 reversed the dysfunction of TILs. **a** IFN-γ production by TILs was determined in TILs or TILs co-cultured with tumor cell lines using an ELISA assay in three independent experiments. The data are represented as the mean ± SD. **b** IFN-γ production by TILs was determined in TILs in which the expression of SRSF2 was inhibited by siSRSF2 or these TILs co-cultured with tumor cell lines using an ELISA assay in three independent experiments. The data are represented as the mean ± SD. **c**–**i** The frequency of IFN-γ + and CD8 + TILs was assessed by flow cytometry in TILs in which the expression of SRSF2 was inhibited by siSRSF2 or these TILs co-cultured with tumor cell lines isolated from Sample 3 (**c**), Sample 4 (**d**), Sample 7 (**e**), Sample 10 (**f**), Sample 11 (**g**), Sample 12 (**h**), and Sample 13 (**i**). *p < 0.01

Studies have reported that TILs exhibited cell exhaustion in most solid tumors [29–31]. To determine the status of TILs in RCC, we isolated TILs and tumor cells from primary RCC specimens, co-cultured these cells, and examined IFN-γ production using an ELISA assay to investigate the direct immune responses against autologous tumor cells in TILs. The results demonstrated that only tumor cell lines isolated from sample 10 could stimulate the production of IFN-γ in TILs (Fig. 1a). However, the downregulation of SRSF2 by SRSF2 siRNA (siSRSF2) (Fig. S1) in these TILs could significantly improve their immune response against tumor cell lines in sample 3, 4, 7, 10, 11, 12, and 13 (Fig. 1b). Moreover, we conducted flow cytometry with antibodies against IFN-γ and CD8 in these positive samples, and found that the frequency of IFN-γ + and CD8 + TILs among the TILs co-cultured with the autologous tumor cell lines was significantly higher than that in the TILs alone, while the expression of SRSF2 in these TILs was downregulated by siSRSF2 (Fig. 1c–i). Taken together, our results show that most TILs isolated from primary RCC specimens could not recognize autologous tumor cells and that SRSF2 functions as an important regulator of the immune response against tumor cells in TILs.

### SRSF2 regulates the expression of multiple immune checkpoints

**Fig. 2:**
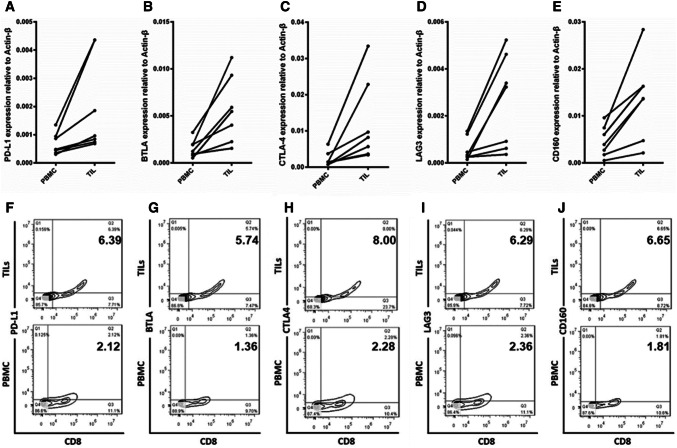
The elevated expression of immune checkpoint molecules in TILs. The expression level of immune checkpoint molecules was determined in TILs and compared with that in autologous PBMCs by real-time PCR (**a**–**e**) and flow cytometry (**f**–**j**)

A previous study reported that immune checkpoints on T cells represent the functional status of these cells [32]. To investigate why the tumor cells could not trigger the immune response in the autologous TILs, we compared the expression level of multiple immune checkpoints between TILs and peripheral blood mononuclear cells (PBMCs) containing many effector T cells. We isolated TILs and PBMCs from tumor tissues and peripheral blood from samples 3, 4, 7, 10, 11, 12, and 13. Then, the total RNA was extracted from these cells and real-time PCR was conducted. Our results showed that the expression of PD-L1, BTLA, CTLA4, LAG3, and CD160 was significantly increased in the TILs compared with that in the PBMCs (Fig. 2a–e). For further confirmation, we performed flow cytometry using antibodies against these immune checkpoints, and CD8 in the TILs and PBMCs from sample 13 that exhibited greater alteration in the immune response following the knockdown of SRSF2. The results showed that the frequency of immune checkpoint + and CD8 + TILs was significantly higher among the TILs than that among the PBMCs (Fig. 2f–j).

**Fig. 3:**
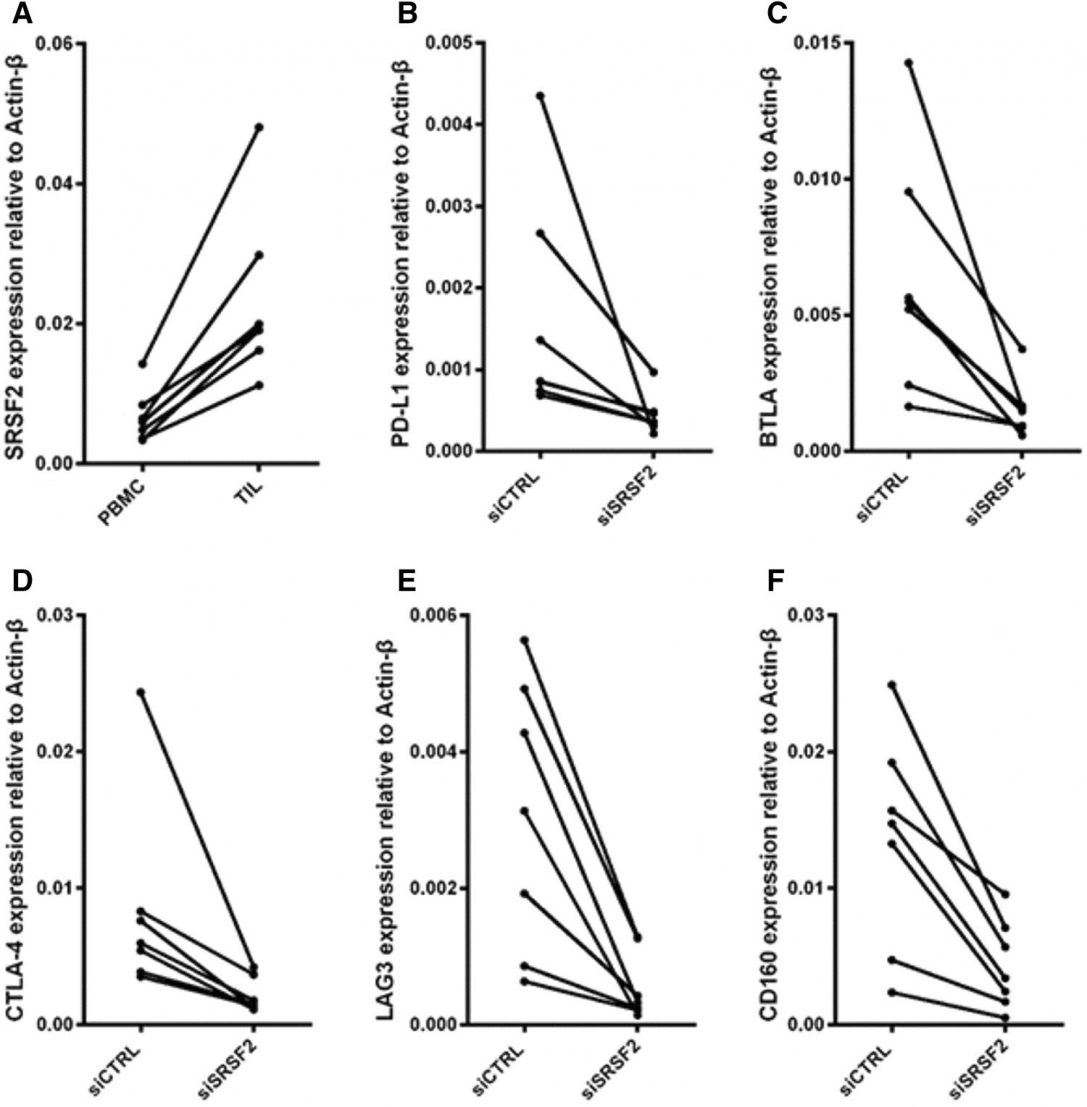
SRSF2 regulates the expression of multiple immune checkpoint genes. **a** The expression level of SRSF2 was determined in TILs isolated from Sample 3, 4, 7, 10, 11, 12, and 13 and compared with that in autologous PBMCs by real-time PCR. **b**–**f** 36 h after these TILs were transfected with SRSF2-targeting siRNAs (siSRSF2) or negative control siRNAs (siCTRL), the expression level of PD-L1 (**b**), BTLA (**c**), CTLA4 (**d**), LAG3 (**e**), and CD160 (**f**) was determined by real-time PCR

To uncover the mechanism by which SRSF2 regulates the immune response of autologous TILs, we first used a RCC data set (GSE40435) from the National Center for Biotechnology Information (NCBI) database to analyze SRSF2 expression levels in the RCC tumor tissue and adjacent tumor tissue. As shown in Figure S2, SRSF2 expression was increased in RCC tumor tissue. Then, we examined the expression level of SRSF2 in TILs and PBMCs, and found that the TILs exhibited a higher level than the autologous PBMCs (Fig. 3a). Thereafter, we elucidated the relationship between the expression of SRSF2 and immune checkpoints. We silenced SRSF2 expression by siSRSF2 and found that the depletion of SRSF2 downregulated the expression of these immune checkpoints (Fig. 3b–f). Taken together, these results demonstrated that TILs isolated from tumor tissues expressed higher levels of multiple immune checkpoints and that SRSF2 regulates the expression of these immune checkpoints.

### SRSF2 regulates the transcriptional activities of immune checkpoint genes

**Fig. 4:**
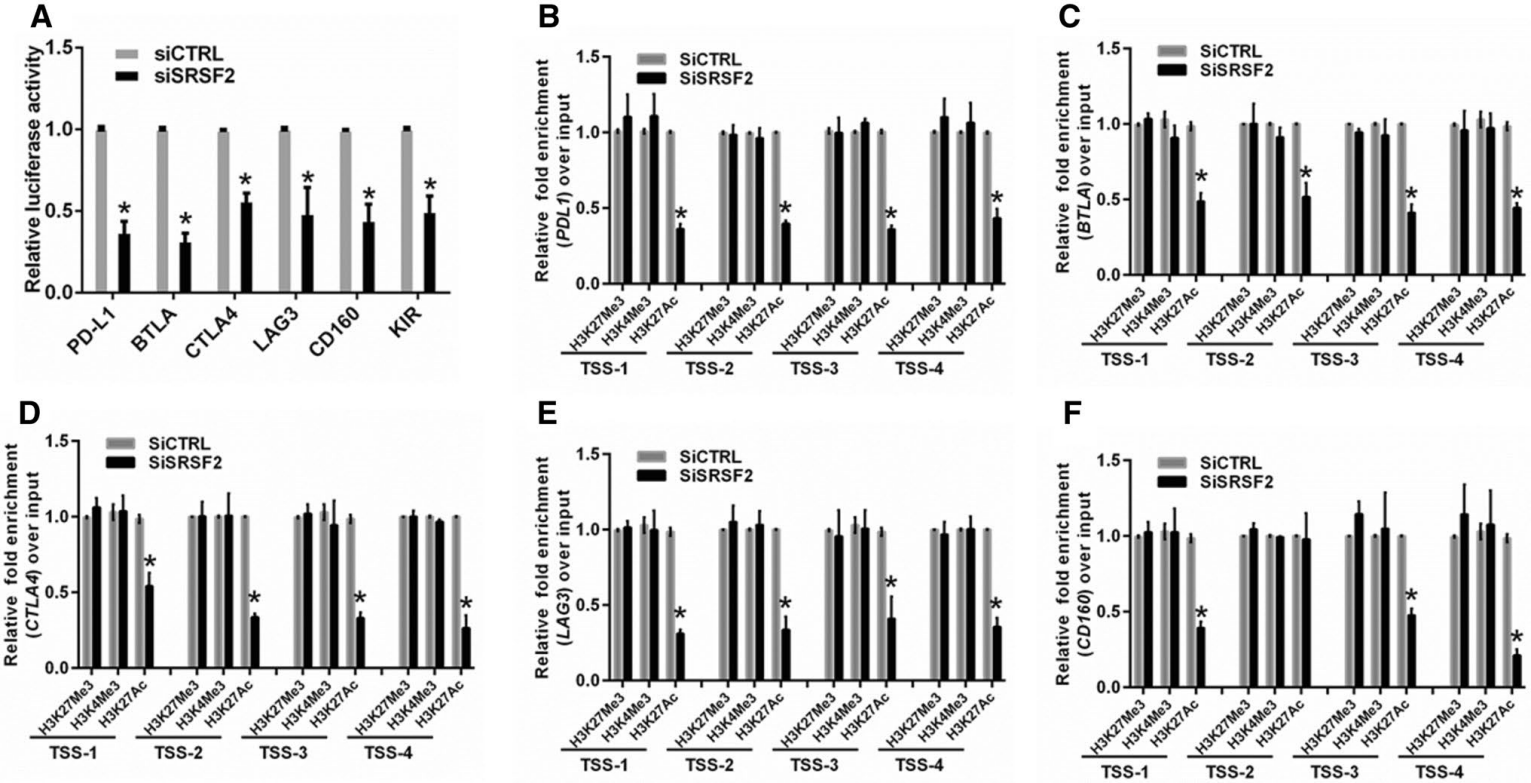
SRSF2 regulates the transcriptional activities of immune checkpoint genes by regulating histone modification. **a** After the cotransfection with the SRSF2 siRNAs or negative control siRNAs and the pGL3 enhancer plasmid containing the *PD*-*L1* promoter, *BTLA* promoter, *CTLA*-*4* promoter, *LAG3* promoter or *CD160* promoter for 36 h, the relative transcriptional activities of theses promoters were determined with a luciferase assay in three independent experiments. The data are represented as the mean ± SD. **b**–**f** Jurkate E6 cells transfected with SRSF2 siRNAs or negative control siRNAs were collected for ChIP assays to analyze the relative fold enrichment of the *PD*-*L1* promoter (**b**), *BTLA* promoter (**c**), *CTLA*-*4* promoter (**d**), *LAG3* promoter (**e**), or *CD160* promoter (**f**) by an anti-H3K4Me3 antibody, anti-H3K27Me3 antibody or anti-H3K27Ac antibody. The data points represent mean values determined from three independent experiments. The data are presented as the mean ± SD. *p < 0.01

To better understand the mechanism by which SRSF2 regulates these related genes, we verified the regulatory roles of SRSF2 in the expression of these immune checkpoint genes in Jurkate E6, which is a human immortalized T-lymphocyte cell line, and obtained results similar to those obtained in the TILs (Fig. S3). Our previous study illustrated that in addition to participating in pre-mRNA splicing, SRSF2 could function as a transcriptional activator of the expression of related genes by binding the promoters of these genes [28]. Therefore, we conducted a luciferase assay to determine whether SRSF2 directly regulates the transcriptional activities of these immune checkpoints. We generated a luciferase reporter construct containing the promoter of this gene. The results showed that the transcriptional activity of the *PD*-*L1*, *BTLA*, *CTLA4*, *LAG3,* and *CD160* gene promoters in the SRSF2-depleted Jurkate E6 cells was inhibited (Fig. 4a). To determine whether the downregulation of SRSF2 alters histone modifications near the transcriptional start sites (TSS) of these genes, we designed sets of primer pairs that recognize the corresponding TSS regions of these genes (Fig. S4) and performed chromatin immunoprecipitation (ChIP) experiments using antibodies against tri-methylated histone H3 at lysine 4 (H3K4Me3), acetylated histone H3 at lysine 27 (H3K27Ac), and tri-methylated histone H3 at lysine 27 (H3K27Me3) in Jurkate E6 cells transfected with SRSF2 siRNAs (siSRSF2) or control siRNAs (siCTRL). In these histone modifications, H3K4Me3 and H3K27Ac at transcription start sites (TSS) serve as markers of actively transcribed genes, while H3K27Me3 at TSS is associated with gene repression [33]. The results showed that knocking down SRSF2 decreased the enrichment of H3K27Ac at the promoters of these genes (Fig. 4b–f). Taken together, our results demonstrated that SRSF2 regulates *PD*-*L1*, *BTLA*, *CTLA4*, *LAG3,* and *CD160* transcriptional activity by altering the histone modification status of these gene promoters.

### SRSF2 is associated with the P300/CBP complex

**Fig. 5:**
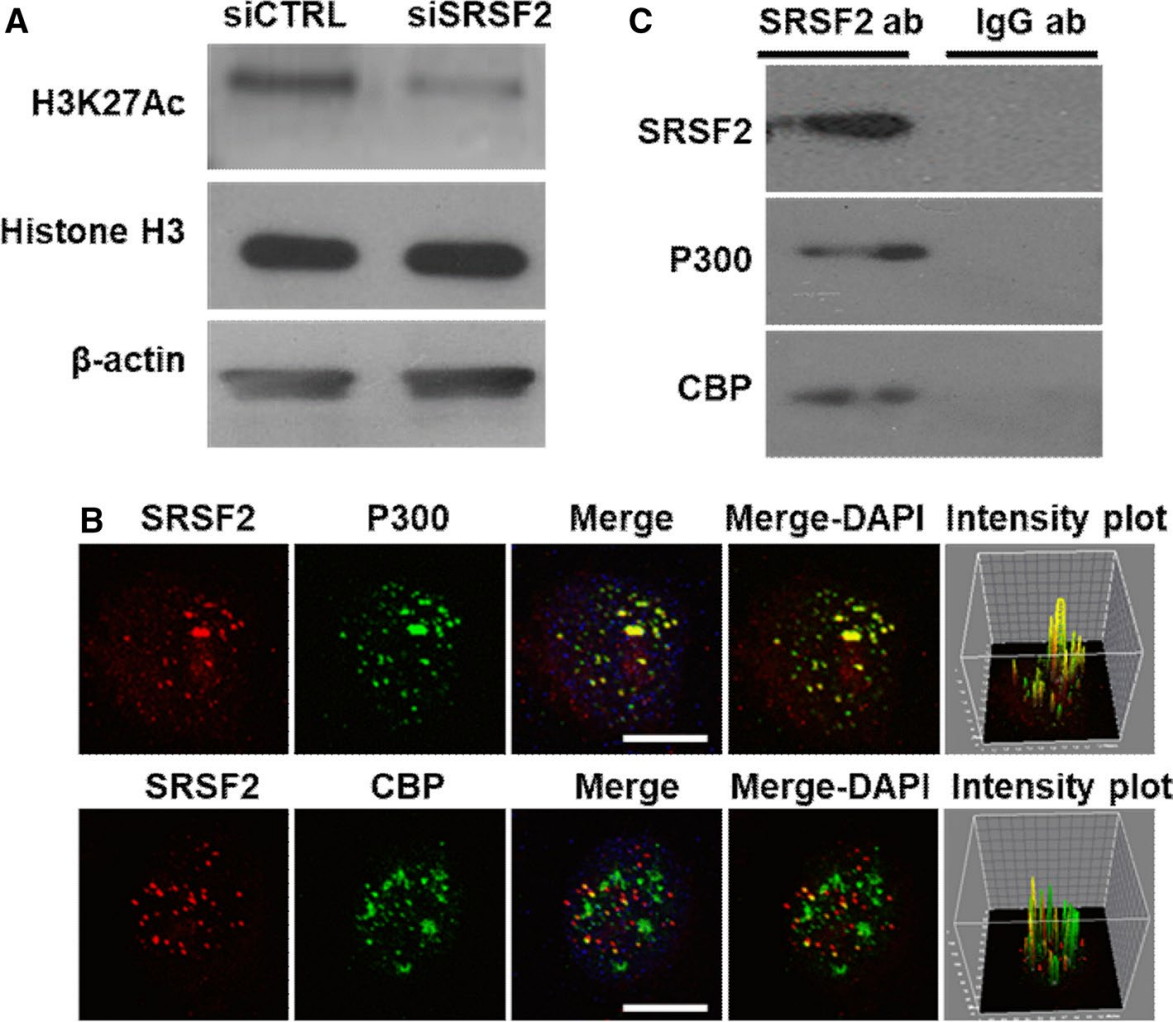
SRSF2 associates with the P300/CBP complex. ** a** Protein levels of H3K27Ac, Histone H3 and β-actin in Jurkate E6 cells transfected with SRSF2 siRNAs or negative control siRNAs were measured by western blotting. **b** Jurkate E6 cells were cultured in poly-d-lysine,coated plates, fixed, incubated with an anti-SRSF2 antibody (red), anti-P300 antibody (green), or anti-CBP antibody (green), and subjected to a confocal analysis. The intensity plots of the red and green channels were analyzed with ImageJ software. Scale bars, 10 µm. **c** Jurkate E6 cells lysates were collected and immuno-precipitated with an anti-SRSF2 antibody or anti-IgG antibody, followed by a western blot analysis of SRSF2, P300, and CBP

To further study the molecular mechanism by which SRSF2 regulates histone acetylation at the promoters of the target genes, we detected and analyzed the effect of SRSF2 on the global levels of H3K27Ac. The results revealed a decrease in H3K27Ac in the SRSF2 knockdown cells (Fig. 5a). Given that histone acetylation requires the acetyl-transferases P300/CBP complex and is involved in the epigenetic regulation of gene expression [34, 35], in our study, we focused on the relationship between SRSF2 and P300/CBP. Since SRSF2 and P300/CBP are considered transcriptional regulators of great magnitude [28, 34] and both regulate histone acetylation, we first conducted immunofluorescence experiments to determine whether SRSF2 is associated with P300/CBP in spatial location. As shown in Fig. 5b, SRSF2 was largely colocalized with P300, and a small amount of SRSF2 was colocalized with CBP. In addition, pixel intensity plots of each merged channel were generated by ImageJ software (Fig. 5b, right panels). Additionally, we performed an immunoprecipitation assay to confirm this association. The Jurkate E6 cell lysates were harvested, and an immunoprecipitation assay was performed using antibodies against SRSF2 or IgG, followed by western blotting using an anti-SRSF2 antibody, anti-P300 antibody and anti-CBP antibody. P300 and CBP were indeed specifically pulled down in the cell lysates (Fig. 5c). These data are the first to show that SRSF2 is colocalized with P300/CBP complex to regulate H3K27 acetylation near the TSS of related genes.

### SRSF2 is required for the recruitment of STAT3 to H3K27Ac

**Fig. 6:**
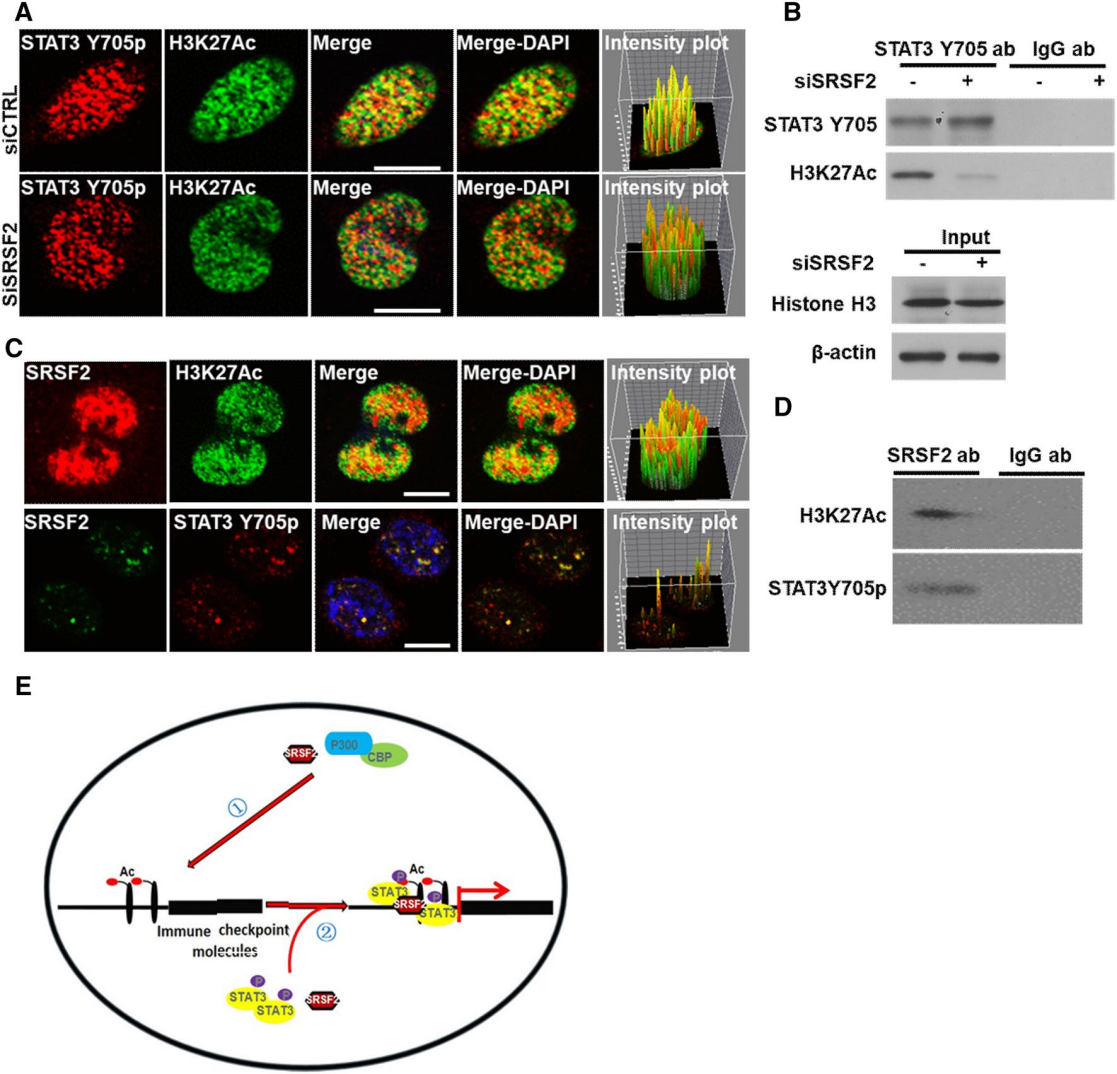
SRSF2 associates with STAT3 and H3K27Ac. **a**. Jurkate E6 cells transfected with SRSF2 siRNAs or negative control siRNAs were cultured in poly-d-lysine-coated plates, fixed, incubated with an anti-STAT3 Y705p antibody (red), and anti-H3K27Ac antibody (green), and subjected to a confocal analysis. The intensity plots of the red and green channels were analyzed with ImageJ software. Scale bars, 10 μm. **b**. Jurkate E6 cell lysates transfected with SRSF2 siRNAs or negative control siRNAs were collected and immuno-precipitated with an anti-STAT3 Y705p antibody or anti-IgG antibody, followed by a western blot analysis of STAT3 Y705p, H3K27Ac, Histone H3, and β-actin. (C). Jurkate E6 cells were cultured in poly-d-lysine-coated plates, fixed, incubated with an anti-SRSF2 antibody (red), anti-H3K27Ac antibody (green) or anti-STAT3 Y705p antibody (green), and subjected to a confocal analysis. The intensity plots of the red and green channels were analyzed with ImageJ software. Scale bars, 10 μm. **d**. Jurkate E6 cell lysates were collected and immuno-precipitated with an anti-SRSF2 antibody or anti-IgG antibody, followed by a western blot analysis of H3K27Ac and STAT3 Y705p. **e**. Schematic model of the roles of SRSF2 in P300/CBP-mediated H3K27 acetylation ① and STAT3 Y705p-mediated gene transcription ②

Given that the H3K27Ac level near the TSS of genes influences the binding ability of STAT3, an important transcriptional factor for gene expression [36], to target genes [37, 38], and that STAT3 plays vital roles in inducing tumor immune tolerance [39], we hypothesized that STAT3 is involved in SRSF2-mediated gene expression immune checkpoints. To achieve it, we analyzed STAT3 expression levels in the RCC tumor tissue and adjacent tumor tissue with the RCC data set (GSE40435), and the results demonstrated that STAT3 expression was significantly increased in RCC tumor tissue (Fig. S5A). Additionally, the general consensus STAT3-binding motif [40] was identified at the TSS of these genes (Figure S5B-F), and silencing STAT3 expression with specific STAT3-targeting siRNAs resulted in reduced levels of the expression of these genes (Fig. S5G). Furthermore, the immunofluorescence and immunoprecipitation results showed that STAT3 Y705p, which is the active form of STAT3, is largely colocalized with H3K27Ac and that the inhibition of endogenous SRSF2 expression could disrupt this association between STAT3 Y705p and H3K27Ac (Fig. 6a, b), suggesting that SRSF2 plays a scaffolding role in the interaction between STAT3 Y705p and H3K27Ac. To clarify the mechanism of this association, we performed immunofluorescence with antibodies against SRSF2, H3K27Ac, and STAT3 Y705p, and immunoprecipitation with antibodies against SRSF2 and IgG. The results demonstrated that SRSF2 interacted with both STAT3 Y705p and H3K27Ac (Fig. 6c, d). Also, a ChIP assay with antibody against to STAT3 Y705 showed that downregulation of SRSF2 resulted in a decrease in the enrichment of STAT3 Y705p bound to the immune checkpoints genes’ promoters (Fig. S6). These results suggested that SRSF2 is required for the recruitment of STAT3 to these immune checkpoint genes.

## Discussion

Since 2013, cancer has become the first cause of death among the urban and rural residents of China. However, the existing treatment methods, such as surgery, radiotherapy, and chemotherapy, cannot effectively cure cancer. Researchers have found that radiotherapy and chemotherapy can cause apoptosis and necrosis in cancer cells. However, these treatments also cause senescence. These senescent cells can promote cancer stemness by activating the Wnt signaling pathway, which can eventually lead to cancer relapse and metastasis [41, 42]. In fact, many tumor-bearing patients naturally develop a complex immune response against tumor activity. However, the effector immune cells in the tumor microenvironment are suppressed by tumor cells and other immunosuppressive cells, such as MDSCs, TAMs, and Tregs.

Growing evidence suggests that developing techniques that inhibit the effects of the tumor microenvironment on immune cells could be helpful in enhancing the anti-tumor efficacy of tumor immunotherapy [43]. In recent years, targeted immune checkpoint molecules have been considered an effective therapeutic strategy for eliminating the tumor microenvironment impacts [44]. In the current study, we found that the inhibition of SRSF2 expression in TILs significantly improves its anti-tumor immune response via the epigenetic regulation of multiple immune checkpoint molecules. SRSF2 associates with the P300/CBP complex to regulate its acetyl-transferases activity and H3K27 acetylation. Then, SRSF2 recruits the transcriptional factor STAT3 to H3K27Ac near the TSS of these immune checkpoint genes to regulate gene transcription (Fig. 6e).

In this study, we first found that most TILs in the RCC specimens are in an immunosuppression status with a high expression of multiple immune checkpoint molecules and could not produce an immune response against autologous tumor cells. To be exciting, we found that blocking SRSF2 expression in TILs could reverse its immunosuppression through the downregulation of the expression level of these immune checkpoint genes. To date, many inhibitors targeting immune checkpoint molecules have been approved for the treatment of solid tumors and displayed certain therapeutic efficacy in preclinical and clinical trials [45–50]. However, due to off-target effects, these immunotherapies have led to many immune-related adverse events [51]. Additionally, immune checkpoint inhibitor treatments are considered dangerous for the treatment of patients who have underlying autoimmune diseases or chronic immunosuppression [52]. All these defects are attributed to the off-target effects of these immune checkpoint inhibitors. Therefore, developing a strategy to directly downregulate the expression of this immune checkpoint in vitro before their expansion with reinfusion into the tumor-bearing patient may be a good choice for avoiding these defects. In this study, we found that TILs transfected with SRSF2-targeting siRNAs could reverse the exhaustion of TILs by downregulating the expression of multiple immune checkpoint genes, including PD-L1, BTLA, CTLA4, LAG3, and CD160.

Studies have shown that exhausted T cells in the tumor microenvironment exhibit distinct epigenetic profiles [53, 54] and that immune checkpoint inhibitors, such as anti-PD-L1 antibodies, could not alter the epigenetic state to reverse the exhaustion of T cells [54]. In the current study, blocking SRSF2 expression with SRSF2-specific siRNAs could downregulate the level of H3K27Ac near multiple immune checkpoint genes via the disruption of the association between SRSF2 and the acetyl-transferase P300/CBP complex. Additionally, we found that the depletion of SRSF2 decreases the enrichment of the transcriptional factor STAT3 to H3K27Ac by interrupting the association between STAT3 and H3K27Ac. Our data indicate that SRSF2 may function as an epigenetic regulator.

Taken together, this study reveals that most of TILs isolated from RCC tissue are exhausted and downregulation of SRSF2 in these TILs could significantly improve their immune response against tumor cells through inhibiting the expression of multiple immune checkpoint molecules via the alteration of the epigenetic status of these genes. All these data suggest that SRSF2 may be a therapeutic target to block tumor-associated suppressive pathways.

## Materials and methods

### Patients and samples

In total, 13 patients with histologically confirmed RCC undergoing partial nephrectomy at the Department of Urology of the First Affiliated Hospital of Shenzhen University Hospital between October 2016 and September 2017 were enrolled in the study. The study was approved by the Ethics Committee of the First Affiliated Hospital of Shenzhen University. All patients signed a written consent form. Tumor specimens of at least 1 cm^3^ were obtained from primary RCC tumors. Blood samples were collected prior to surgery, and peripheral blood mononuclear cells (PBMCs) were isolated from the blood samples.

### Generation of TILs and tumor cells and cell culture

The generation of TILs was performed according to Dudley’s protocol [55]. In brief, a single tumor fragment was placed in individual wells of a 24-well tissue culture plate with 2 mL of complete medium (CM) plus 6000 IU per mL of rhIL-2 (Chiron Corp., Emeryville, CA). The CM consisted of RPMI 1640 (Life Technologies, 21875091), 25 mmol/L HEPES pH 7.2 (Life Technologies, 15630080), 10 U/ml penicillin–streptomycin (Life Technologies, 15140163), and 2 mmol/L-glutamine (Life Technologies, 25030081), supplemented with 10% human serum (Sigma-Aldrich, H4522). The plates were placed in a humidified 37 °C incubator with 5% CO2. Half of the medium was changed at day 5 and thereafter every 2–3 days thereafter.

For generation of tumor cells, Single-cell suspensions were obtained from minced tumor by enzymatic digestion at 37 °C in 5 ml of DMEM/F-12 (Life Technologies, 11320033) supplemented with 5 mg/mL collagenase type II (Life Technologies, 17101015), 30 U/ml DNAse I (Sigma-Aldrich, D5025), and 10 μM Y-27632 dihydrochloride (AbMole, M1817). After 1 h for digestion, the tumor cell suspensions were centrifuged, resuspended, and filtered through 40 µm cell strainer (Sigma-Aldrich, CLS431750-50EA) and then were cultured in DMEM/F12 supplemented with 20% FBS (Gibco, 10270), 1% Gluta-max (Life Technologies, 35050038), 1% HEPES, 5 ng/mL FGF2 (Peprotech, 100-18B), 5 ng/mL EGF (Peprotech, AF-100-15), 5 mg/L insulin (Sigma-Aldrich, I9278), 5 mg/L transferrin (Sigma-Aldrich, T3309), 25 μg/L hydrocortisone (Sigma-Aldrich, H0888), and 10 μM Y-27632 dihydrochloride (AbMole, M1817) in a humidified 5% CO2 incubator at 37 °C.

Jurkate E6 cells (American Type Culture Collection, ATCC) were grown in RPMI 1640 media supplemented with l-glutamine, 10% fetal bovine serum (PAA, A15-101), and 1% Penicillin/Streptomycin (Gibco/Invitrogen Ltd, 15140-122) in a humidified 5% CO2 incubator at 37 °C.

### Data set

A RCC data set (GSE40435) was downloaded from Gene Expression Omnibus (GEO, https://www.ncbi.nlm.nih.gov/gds/) database. The expression of SRSF2 and STAT3 in the RCC tumor tissue and adjacent tumor tissue was analyzed with nonparametric Kolmogorov–Smirnov test.

### Cell transfection, RNA isolation, reverse transcription, and qPCR

All of the synthetic siRNAs and the negative control (siCTRL) siRNA were purchased from Shanghai GenePharma Co., Ltd. All the siRNAs were transfected with Neon^®^ Transfection System (Invitrogen, MPK5000) according to the manufacturer’s protocol. The sequences of the siRNAs used are listed in Table S1. Total RNA was isolated with RNAiso Plus (Takara, D9108B) according to the manufacturer’s protocol. Real-time PCR was performed with ReverTra Ace^®^ qPCR RT Master Mix with gDNA remover (Toyobo, FSQ-301) and SYBR Green PCR Master Mix (Toyobo, QPK-201). All mRNA levels were measured and normalized to that of β-actin mRNA. The primers used are listed in Table S1.

### Western blotting

Cells were lysed in ice-cold whole-cell extract buffer B (50 mM Tris–HCl [pH 8.0], 4 M urea, and 1% Triton X-100) supplemented with complete protease inhibitor cocktail (Roche). The cell extracts were resolved with SDS-PAGE and analyzed with western blotting. The protein bands were visualized with ECL Blotting Detection Reagents. The antibodies used for western blotting included an anti-SRSF2 antibody (Santa Cruz Biotechnology, sc-10252), anti-H3K27Ac antibody (Abcam, ab4729), anti-Histone H3 antibody (Abcam, ab1791), and anti-β-actin antibody (Proteintech, 60008-1-Ig).

### Flow cytometry

To investigate the functions of SRSF2 in the immune response of TILs against autologous tumor cell lines, TILs transfected with SRSF2 siRNA were co-cultured with autologous tumor cell lines. Then, flow cytometry was performed with an anti-CD8 antibody (Biolegend, 344704) and anti-IFN-γ antibody (BD Biosciences, 554552) on BD AccuriC6 (BD Biosciences). To examine the expression level of the immune checkpoints in the TILs and PBMCs, the frequency of immune checkpoint + and CD8 + TILs was assessed using flow cytometry on BD AccuriC6 (BD Biosciences). The antibodies against the immune checkpoints used for flow cytometry included PD-L1 (BD Biosciences, 561787), BTLA (BD Biosciences, 564802), CTLA-4 (BD Biosciences, 561717), LAG3 (BD Biosciences, 565617), and CD160 (BD Biosciences, 562351). All data were analyzed by FlowJo software.

### Luciferase assay

For generation of luciferase reporters for promoter assay, three luciferase reporter constructs that inserted the sequences from − 500 bp to + 500 bp relative to the TSS of PD-L1, BTLA, CTLA-4, LAG3 and CD160 were purchased from Shanghai GenePharma Co. Ltd. Luciferase activities were assayed using a Dual-Luciferase Reporter System (Promega, E1960) according to the manufacturer’s protocol.

### ChIP assay

To investigate the roles of SRSF2 in the status of histone modification near the TSS of genes, ChIP assay was conducted according to Dahl’s protocol [56]. In brief, 1 × 10^6^ Jurkate E6 cells transfected with SRSF2 siRNA or control siRNAs were fixed with 1% formaldehyde and sonicated on ice for 10x10 s, with 20 s pauses between each 10 s session and 30% power using a probe sonicator (Sonics & Materials, Inc.). After centrifugation, the supernatants were incubated with anti-H3K4Me3 antibody (Abcam, ab8580), anti-H3K27Me3 antibody (Abcam, ab6002), anti-H3K27Ac antibody (Abcam, ab4729), or anti-STAT3 Y705 antibody (Abcam, ab76315). Chromatin DNA was purified by Dynabeads protein G (Invitrogen, 10004D) and subjected to real-time PCR. The region-specific primers used are listed in Table S1.

### Immunoprecipitation assay

The immunoprecipitation assay was performed using the Immunoprecipitation Protein G Dynabeads^®^ kit (Invitrogen, 10007D) according to the manufacturer’s protocol. The antibodies used for immunoprecipitation included anti-SRSF2 antibody (Abcam, ab11826), anti-P300 antibody (Abcam, ab59240), anti-CBP antibody (Abcam, ab50702), anti-STAT3Y705p antibody (Abcam, ab76315), and anti-H3K27AC antibody (Abcam, ab4729).

### Immunofluorescence microscopy

To determine the relationship between SRSF2 and P300/CBP, the Jurkate E6 were cultured in Poly-d-lysine-coated plates, fixed with 4% paraformaldehyde, and incubated with anti-SRSF2 antibody (Abcam, ab11826), anti-P300 antibody (Abcam, ab59240), or anti-CBP antibody (Abcam, ab50702) for 1.5 h at room temperature. After the cells were washed and incubated with the secondary antibody, they were counterstained with DAPI and mounted for observation. Cell images were obtained with an Olympus FV1000 confocal microscope. To study the role of SRSF2 in the interaction between STAT3 Y705p and H3K27Ac, the Jurkate E6 transfected with SRSF2 siRNA or negative control siRNA were cultured in Poly-d-lysine-coated plates, fixed with 4% paraformaldehyde, and incubated with the anti-STAT3 Y705 antibody (Abcam, ab76315) and anti-H3K27Ac antibody (Abcam, ab4729) for 1.5 h at room temperature. After the cells were washed and incubated with the secondary antibody, they were counterstained with DAPI and mounted for observation. Cell images were obtained with an Olympus FV1000 confocal microscope. To determine the relationship between SRSF2, STAT3 Y705p, and H3K27Ac, the Jurkate E6 were cultured in Poly-d-lysine-coated plates, fixed with 4% paraformaldehyde, and incubated with anti-SRSF2 antibody (Abcam, ab11826), anti-STAT3 Y705 antibody (Abcam, ab76315), or anti-H3K27Ac antibody (Abcam, ab4729) for 1.5 h at room temperature. After the cells were washed and incubated with the secondary antibody, they were counterstained with DAPI and mounted for observation. Cell images were obtained with an Olympus FV1000 confocal microscope.

### Statistics

Data are expressed as mean ± SD with repeated three times (*p < 0.001). Comparisons between two groups were evaluated using a two-sample *t* test. For three or more groups, standard one-way analysis of variance (ANOVA) with Bonferroni’s test was conducted. A two-tailed probability value < 0.05 was considered to be statistically significant.

## Electronic supplementary material

Below is the link to the electronic supplementary material.

Supplementary material 1 (DOCX 2495 kb)
