# Supplementary material for: Modulation of SRSF2 expression reverses the exhaustion of TILs via the epigenetic regulation of immune checkpoint molecules
Source: Cell Mol Life Sci. 2019 Dec 14;77(17):3441–52. doi: 10.1007/s00018-019-03362-4 (PMC7426320; doi:10.1007/s00018-019-03362-4)
Supplement: Supplementary file 1 — Supplementary material 1 (DOCX 2495 kb) [file 18_2019_3362_MOESM1_ESM.docx]

**Modulation of SRSF2 expression reverses the exhaustion of TILs via the epigenetic regulation of immune checkpoint molecules**

Ziqiang Wang^1,2†^, Kun Li^3†^, Wei Chen^1,2†^, Xiaoxia Wang^1,2^, Yikun Huang^1,2^, Weiming Wang^1,2^, Wanjun Wu^1,2^, Zhiming Cai^1,2*^, Weiren Huang^1,2*^

^1^Department of Urology, Shenzhen Second People’s Hospital, The First Affiliated Hospital of Shenzhen University, International Cancer Center, Shenzhen University School of Medicine, Shenzhen 518039, China;

^2^Guangdong Key Laboratory of Systems Biology and Synthetic Biology for Urogenital Tumors, Shenzhen 518035, China;

^3^Department of Nuclear Medicine, Shandong Provincial Qianfoshan Hospital, the First Hospital Affiliated with Shandong First Medical University, Jinan 250014, China.

†These authors contributed equally to this work.

*To whom correspondence should be addressed to Weiren Huang. Email: pony8980@163.com, Correspondence may also be addressed to Zhiming Cai. Email: caizhiming2000@163.com.

*Corresponding author, Department of Urology, Shenzhen Second People’s Hospital, The First Affiliated Hospital of Shenzhen University, International Cancer Center, Shenzhen University School of Medicine, Shenzhen, China, phone: (86) 755-26036884, fax: (86) 755-26036884.

The authors have declared that no conflict of interest exists.

**Supplementary Materials**

**Supplementary figures**

**
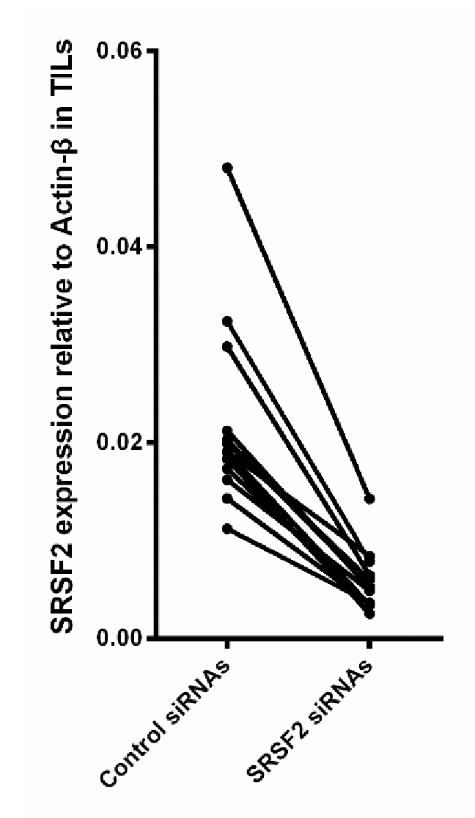
**

**Figure S1. Expression of SRSF2 is downregulated by SRSF2-targeting siRNAs.** TILs were transfected with SRSF2-targeting siRNAs (siSRSF2) or negative control siRNAs (siCTRL) for 36 hours. The levels of SRSF2 were determined with real-time PCR. The data were normalized to the control level.


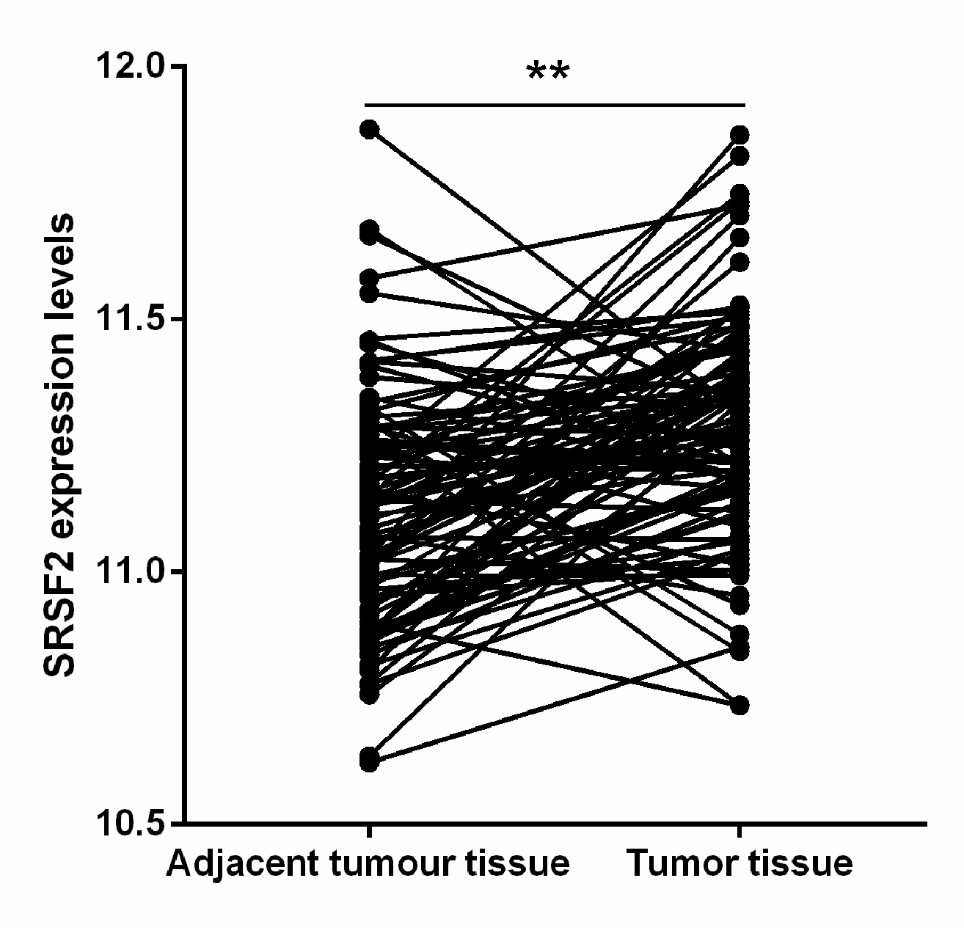


**Figure S2. Expression of SRSF2 is upregulated in RCC tumor tissue.** SRSF2 analysis in the RCC tumor tissue and adjacent tumor tissue in a RCC Dataset (GSE40435). P-values were calculated using nonparametric Kolmogorov-Smirnov tests. **p < 0.0001.

**
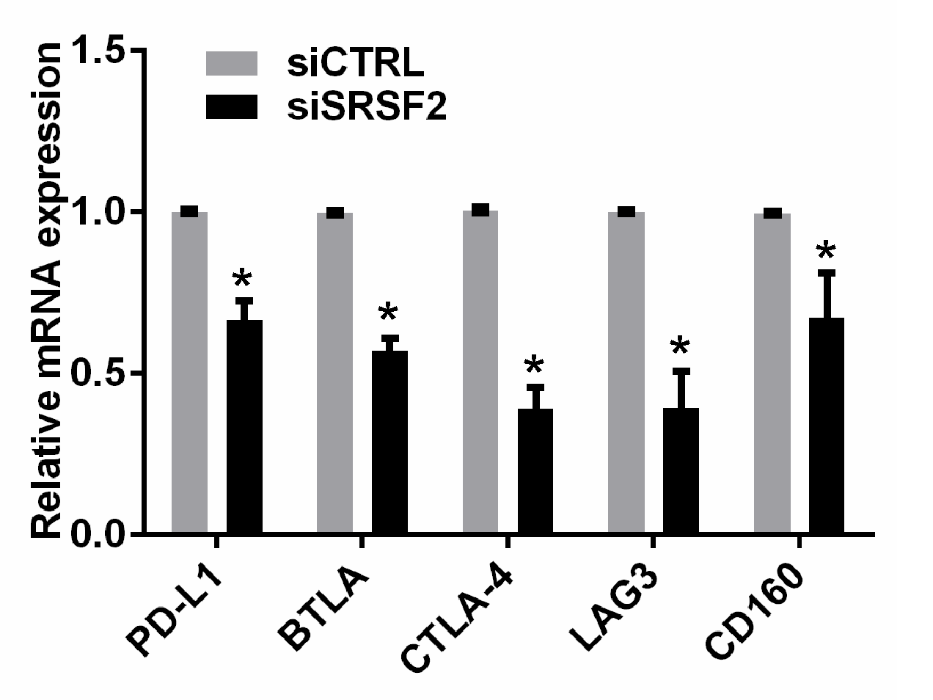
**

**Figure S3. Expression of immune checkpoint molecules regulated by SRSF2.** Jurkate E6 cells were transfected with SRSF2-targeting siRNAs (siSRSF2) or negative control siRNAs (siCTRL) for 36 hours. The levels of the immune checkpoint molecules were determined with real-time PCR in three independent experiments. The data are represented as the mean ± SD. The data were normalized to the control level. *p < 0.01.


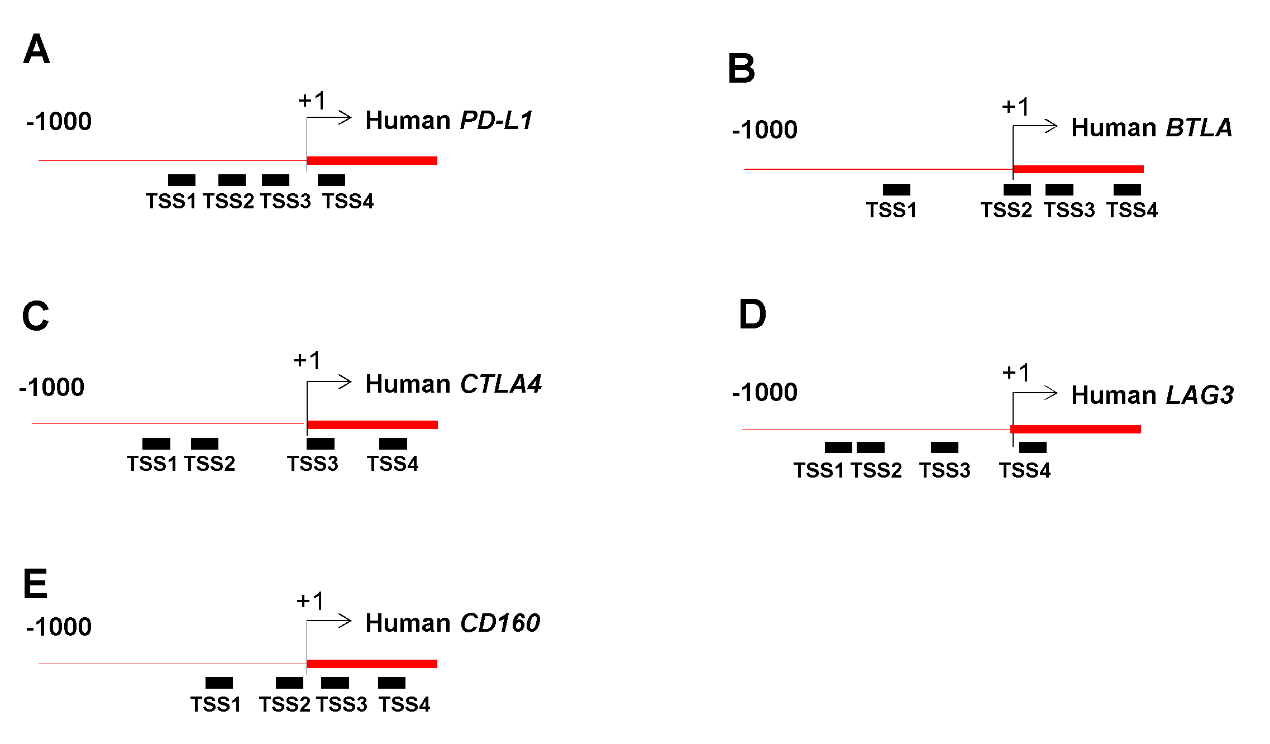


**Figure S4.** Schematic diagram showing the gene structure of *PD-L1*, *BTLA*, *CTLA4*, *LAG3* and *CD160* in which the black boxes represent the primer-amplified regions.


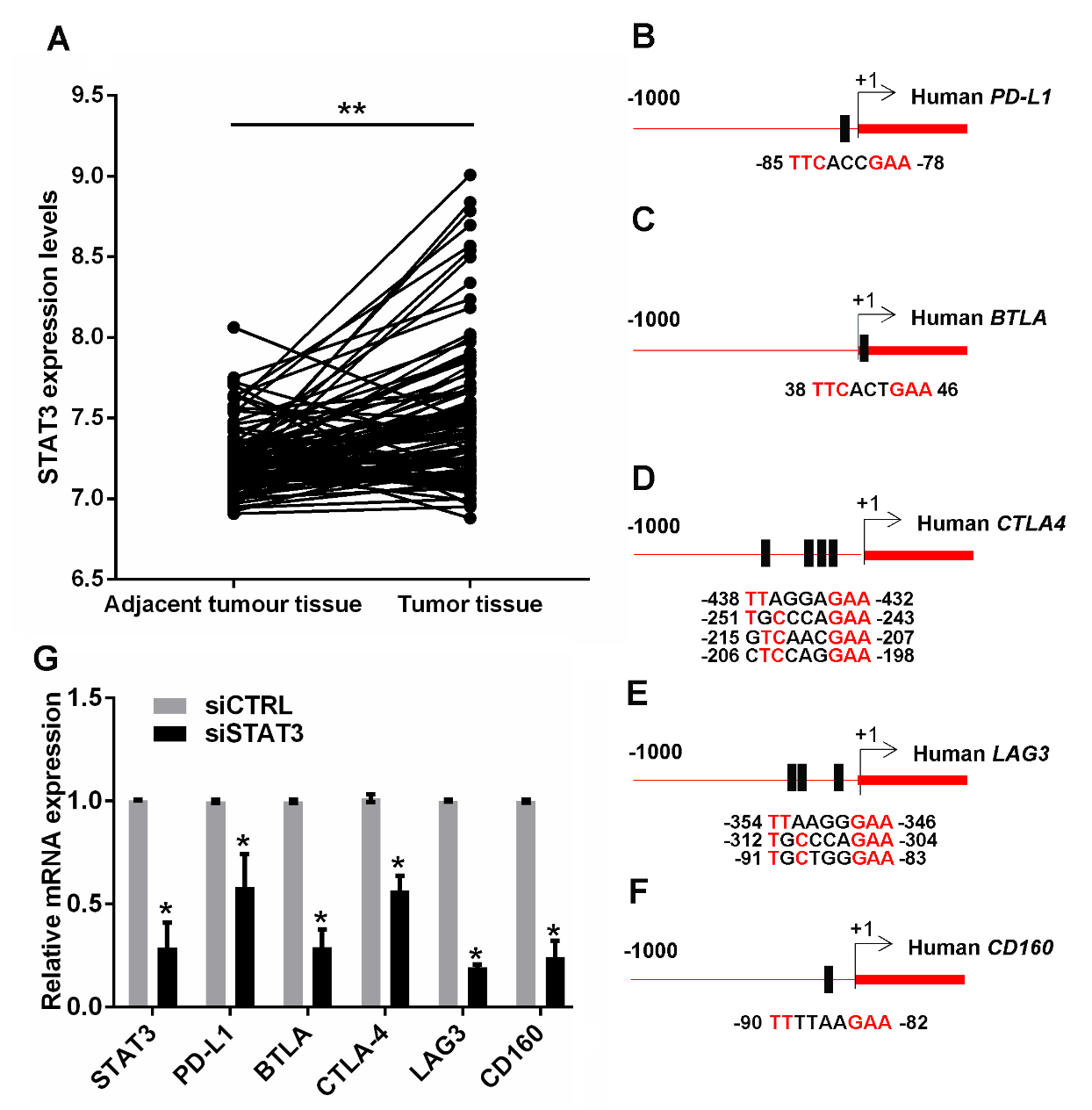


**Figure S5. STAT3 regulates the expression of immune checkpoint molecules.** (A) STAT3 analysis in the RCC tumor tissue and adjacent tumor tissue in a RCC Dataset (GSE40435). P-values were calculated using nonparametric Kolmogorov-Smirnov tests. **p < 0.0001. (B-F). Schematic representation of the STAT3-binding site in the human *PD-L1* (B), *BTLA* (C), *CTLA4* (D), *LAG3* (E) and *CD160* (F) genes. The black box shows the potential binding site, and the red characters indicate matching sequences. (G). Jurkate E6 cells were transfected with STAT3-targeting siRNAs (siSTAT3) or negative control siRNAs (siCTRL) for 36 hours. The levels of the immune checkpoint molecules were determined with real-time PCR in three independent experiments. The data are represented as the mean ± SD. The data were normalized to the control level. *p < 0.01. **p < 0.0001.


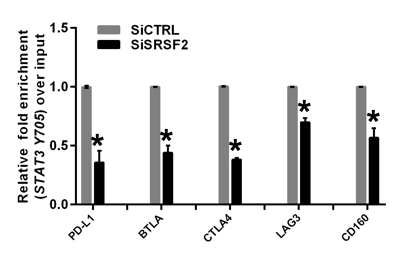


**Figure S6. SRSF2 recruits STAT3 to immune checkpoint genes.** Jurkate E6 cells transfected with SRSF2 siRNAs or negative control siRNAs were collected for ChIP assays to analyze the relative fold enrichment of the *PD-L1* promoter, *BTLA* promoter, *CTLA-4* promoter, *LAG3* promoter or *CD160* promoter by an anti-STAT3 Y705 antibody. The data points represent mean values determined from three independent experiments. The data are presented as the mean ± SD. *p < 0.01.

**Table S1. Sequences of primers and siRNAs used in this study**

**Name sense sequence antisense sequence**

| \| **SiRNAs** \| \| \|  \| \| --- \| --- \| --- \| --- \| \| SRSF2 siRNA \| GUGAGAAGUUGCUUAGAAA \| UUUCUAAGCAACUUCUCAC \|  \| \| STAT3 siRNA \| GAAGGAGGCGUCACUUUCA \| UGAAAGUGACGCCUCCUUC \|  \| \| Negative control siRNA \| UUCUCCGAACGUGUCACGU \| ACGUGACACGUUCGGAGAA \|  \| \| **Primers pairs for Real Time PCR** \| \| \| \| \| SRSF2 \| GAGAACCAAAGGGAGGGGTG \| TGCTGCGTATGCAAGTCTGA \|  \| \| STAT3 \| TGTGTGACACCATTCATTGATGC \| TCCTCACATGGGGGAGGTAG \|  \| \| PD-L1 \| TACTGGCATTTGCTGAACGC \| ACAATTAGTGCAGCCAGGTCT \|  \| \| BTLA \| GAAGCAAGCACCAGGCAAAA \| CTTGCCAGTCTTGAGTTCGG \|  \| \| CTLA-4 \| AGGTGACTGAAGTCTGTGCG \| CATGAGCTCCACCTTGCAGA \|  \| \| LAG3 \| TGGGACCTACACCTGCCATA \| GAGCTCCACACAAAGCGTTC \|  \| \| CD160 \| GGGAACTACACAGTGACGGG \| AGTTTCTTTTGGCACAAGGCT \|  \| \| Actin-beta \| TGACGTGGACATCCGCAAAG \| CTGGAAGGTGGACAGCGAGG \|  \| \| **Primers pairs for ChIP** \|  \|  \|  \| \| PD-L1-TSS1 \| CGAGGAACTTTGAGGAAGTCACA \| AGCCTCTTCAAGGTGACTGAAC \|  \| \| PD-L1-TSS2 \| CAAGGTGCGTTCAGATGTTGG \| TTTCACCGGGAAGAGTTTCG \|  \| \| PD-L1-TSS3 \| AACTGAAAGCTTCCGCCGAT \| CAGCTGCTCAGCGTTGC \|  \| \| PD-L1-TSS4 \| AGGTAGGGAGCGTTGTTCCT \| TACTGCCCCCTAGACCATCG \|  \| \| BTLA-TSS1 \| TGCAGTTGTTGTCTCGATGAAG \| GTGGATGTGGAGAAAACAGTCC \|  \| \| BTLA-TSS2 \| GGTTGAGGTTTTTCCTGCACTC \| TCACAAGCTATCACAAAGGGG \|  \| \| BTLA-TSS3 \| TTTGCTTCTTGTCAGGTTGGC \| TGGGGCAAAAACGTGGTAGA \|  \| \| BTLA-TSS4 \| CAGGTGCCTTACCATGGATGT \| CTGCAGCACTCAGAAGACGA \|  \| \| CTLA-4-TSS1 \| TGGGTTGGCTTTTCTTTGGAC \| GCCTCTGGTTTTGCAGAAGG \|  \| \| CTLA-4-TSS2 \| AGAGGCAGCTTCTTTTCCGC \| CCTTGGGCTAATGGCAGGATT \|  \| \| CTLA-4-TSS3 \| CCACGGCTTCCTTTCTCGT \| GGAGCGGTGTTCAGGTCTTC \|  \| \| CTLA-4-TSS4 \| TCAGCAGTCAAAGGCAGTGA \| AGGGTCTGAAATTGCCTGTGT \|  \| \| LAG3-TSS1 \| GCATGGGGACCTGAGTTGG \| AGTGTACCTGTGTGCGTGAC \|  \| \| LAG3-TSS2 \| CAGCTTTTATCTTCACGCTCCC \| AAGCCTTACTGCACCCCAAA \|  \| \| LAG3-TSS3 \| GTTCCAGGCAGAAATGGTTCG \| TCGCAGTGGAAAGTCTGAGG \|  \| \| LAG3-TSS4 \| GAGACCAGCAGAACGGCAT \| GGCCGGAATCCAGAGGAAAG \|  \| \| CD160-TSS1 \| GGGTGGGAAGATAAGGTCAGTC \| GAAAGGTACCAAGTCTTCTCCTGA \|  \| \| CD160-TSS2 \| CAAAATCTTTGTTCCCCAGGGT \| TAGACTAGGGTGCTGAGGGG \|  \| \| CD160-TSS3 \| GGGTGGCGGAAGATCTATGAC \| CTGGGGGCTCACGTTGAAAT \|  \| \| CD160-TSS4 \| CAGGTGAAGGGATGGGATGG \| TCCAGAGGGCAGAGGACATT \|  \| \|  \|  \|  \|  \| \|  \|  \|  \|  \| |  |  |
| --- | --- | --- | --- | --- | --- | --- | --- | --- | --- | --- | --- | --- | --- | --- | --- | --- | --- | --- | --- | --- | --- | --- | --- | --- | --- | --- | --- | --- | --- | --- | --- | --- | --- | --- | --- | --- | --- | --- | --- | --- | --- | --- | --- | --- | --- | --- | --- | --- | --- | --- | --- | --- | --- | --- | --- | --- | --- | --- | --- | --- | --- | --- | --- | --- | --- | --- | --- | --- | --- | --- | --- | --- | --- | --- | --- | --- | --- | --- | --- | --- | --- | --- | --- | --- | --- | --- | --- | --- | --- | --- | --- | --- | --- | --- | --- | --- | --- | --- | --- | --- | --- | --- | --- | --- | --- | --- | --- | --- | --- | --- | --- | --- | --- | --- | --- | --- | --- | --- | --- | --- | --- | --- | --- | --- | --- | --- | --- | --- | --- | --- | --- | --- | --- | --- | --- | --- | --- | --- | --- | --- | --- | --- | --- | --- | --- | --- |

**Figures for response to comments**

A B


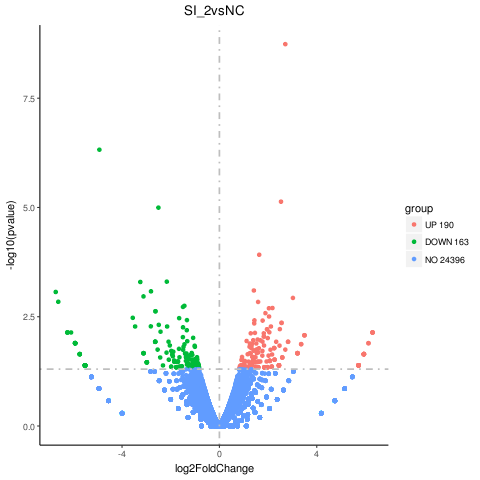

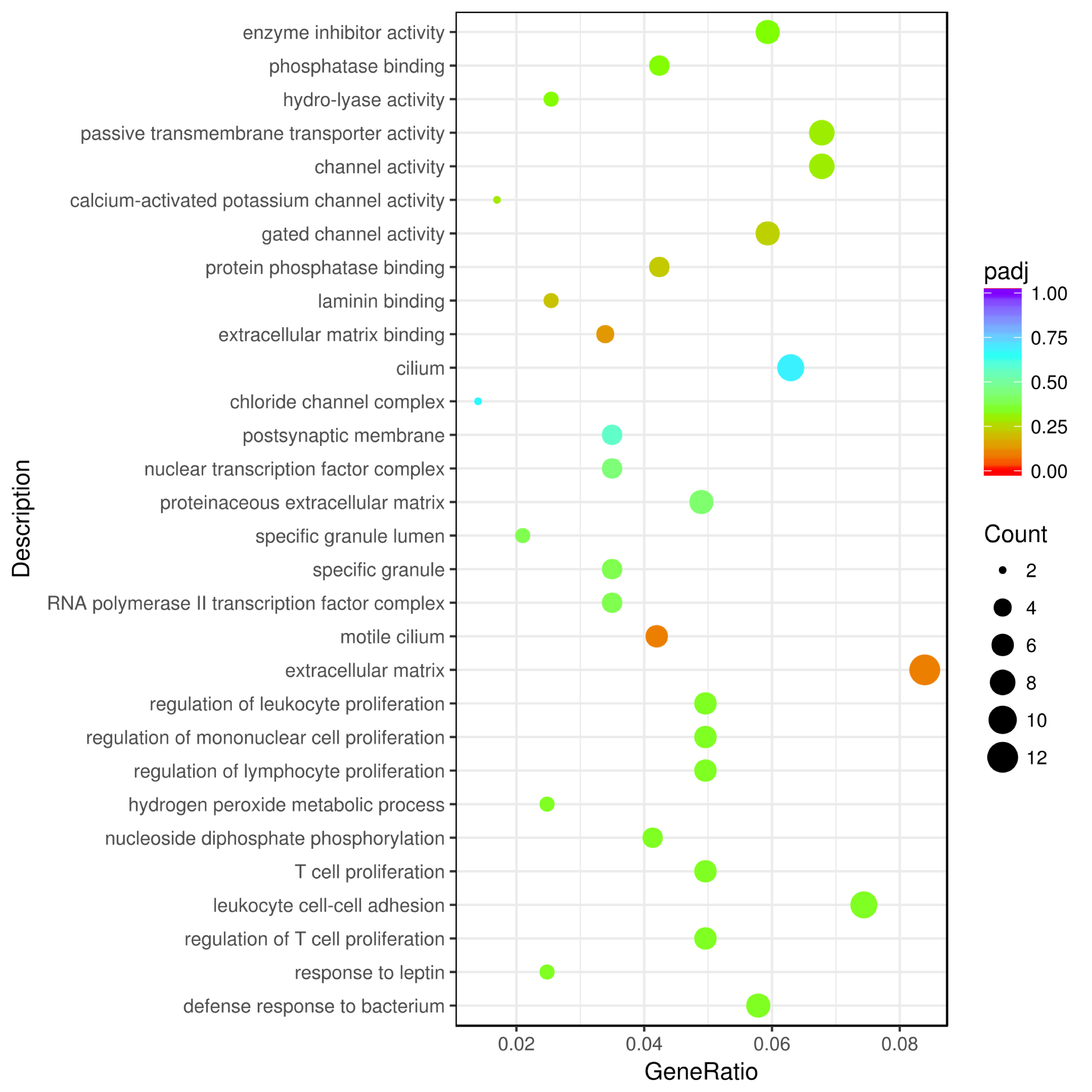


C


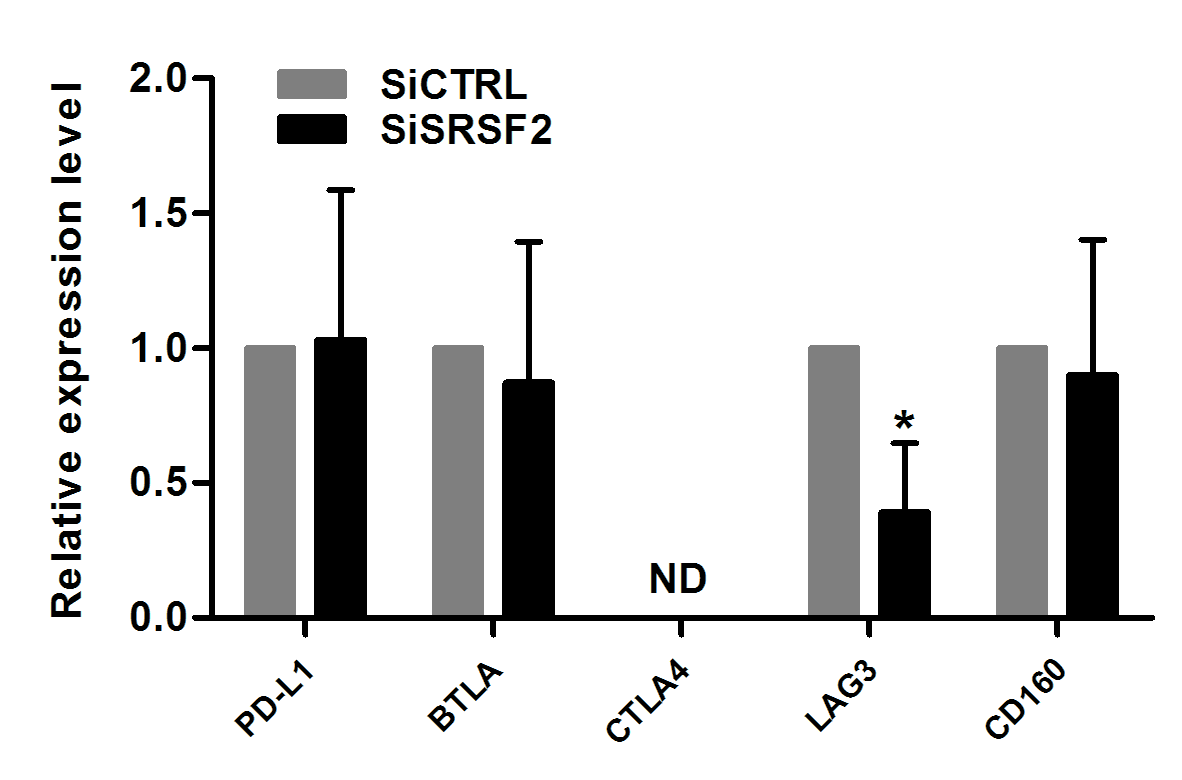


Figure R1. The RNA-seq data of TILs with the knockdown of SRSF2. The number of genes regulated by SRSF2 (A), pathways that these altered genes participated in (B), mRNA levels of PD-L1, BTLA, CTLA4, LAG3 and CD160 (C) were analyzed with the RNA-seq data. ND: not detected. *p < 0.001.

A B


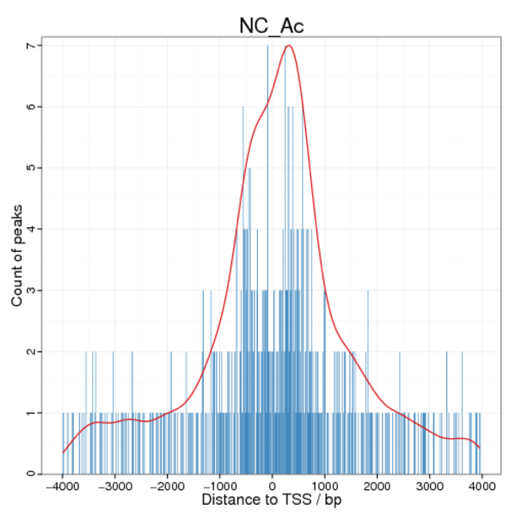

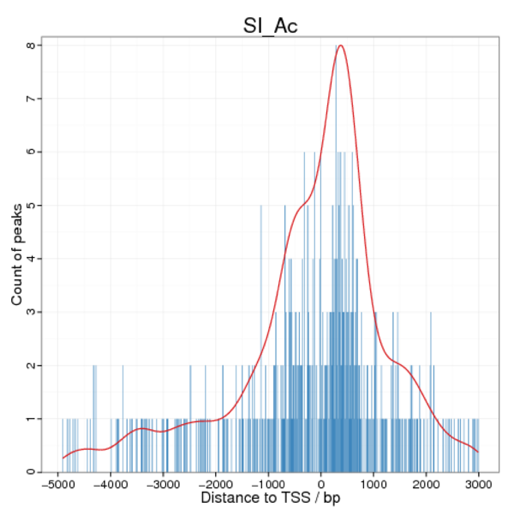


C


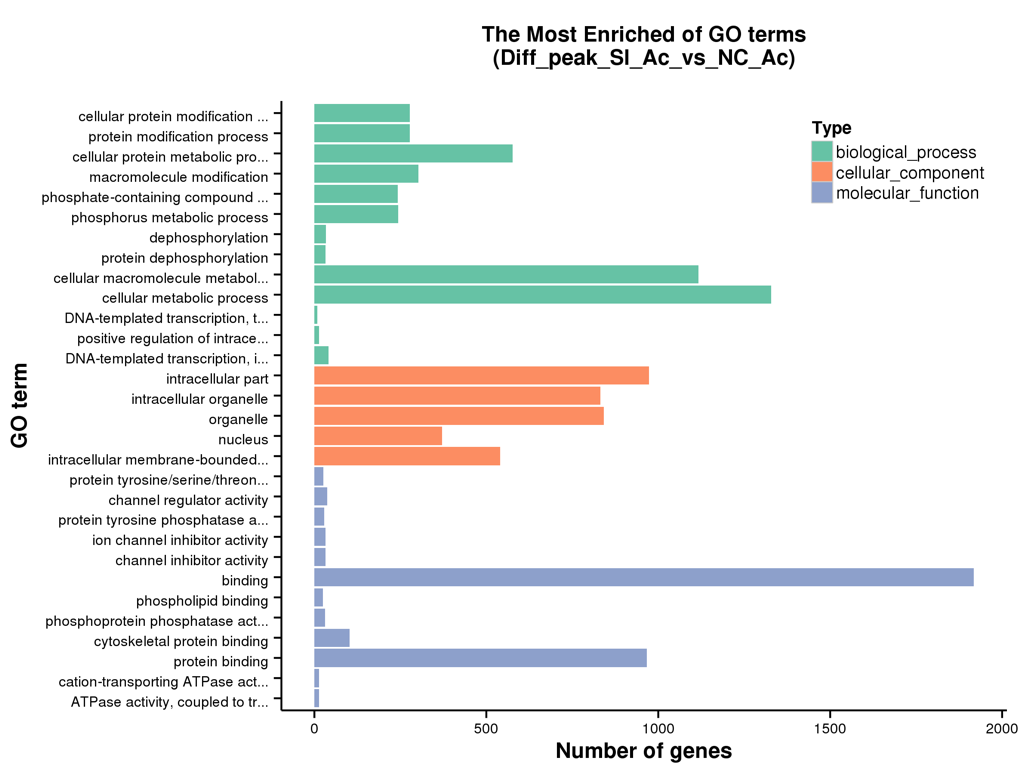


Figure R2. The ChIP-seq data of TILs with the knockdown of SRSF2. The location (A and B) of H3K27Ac and the functions (C) of genes that differentially enriched by H3K27Ac were analyzed with the ChIP-seq data.

**
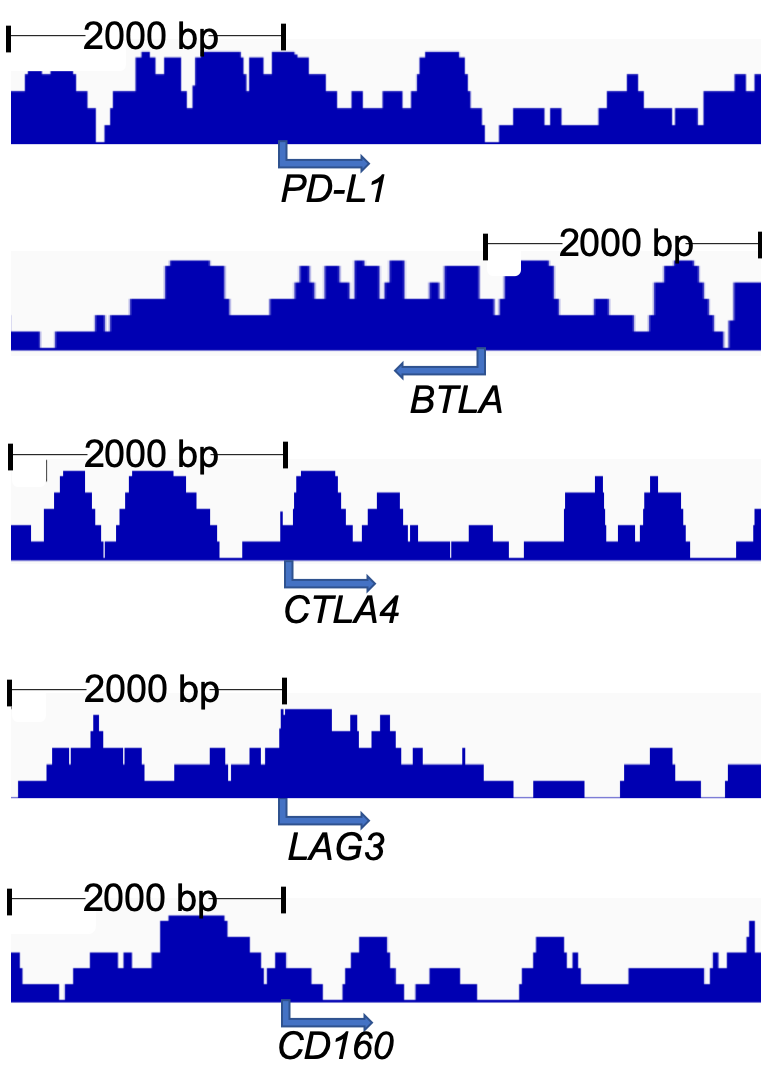
**

Figure R3. Schematic diagram showing H3K27Ac profiles at checkpoints gene promoters (2000 bp upstream from genes TSS).

**Table R1. The list of genes modulated by SRSF2 from RNA-seq and ChIP-seq data**

**ID Gene name Log2 (Fold Change) P-value**

| ENSG00000240303 | ACAD11 | 5.843243093 | 0.02267682 |
| --- | --- | --- | --- |
| ENSG00000070985 | TRPM5 | 5.625032847 | 0.04096458 |
| ENSG00000118997 | DNAH7 | 3.189627466 | 0.02156655 |
| ENSG00000184984 | CHRM5 | 2.408533329 | 0.04103203 |
| ENSG00000228237 | EFCAB14-AS1 | 2.370499004 | 0.00288962 |
| ENSG00000153093 | ACOXL | 2.295498542 | 0.02095211 |
| ENSG00000272980 | Z94721.2 | 2.13073634 | 0.00973269 |
| ENSG00000257488 | LINC02354 | 2.090867599 | 0.04399131 |
| ENSG00000272752 | STAG3L5P-PVRIG2P-PILRB | 2.074241373 | 0.02307085 |
| ENSG00000261460 | AC009690.2 | 1.843026789 | 0.03337634 |
| ENSG00000203799 | CCDC162P | 1.594955698 | 0.01753907 |
| ENSG00000131379 | C3orf20 | 1.550730465 | 0.02169529 |
| ENSG00000145864 | GABRB2 | 1.521740665 | 0.00878145 |
| ENSG00000168610 | STAT3 | 1.368521741 | 0.00115131 |
| ENSG00000272733 | AP000345.2 | 1.355796751 | 0.00783513 |
| ENSG00000177311 | ZBTB38 | 1.193438345 | 0.03728125 |
| ENSG00000183091 | NEB | 1.123376382 | 0.03526717 |
| ENSG00000266969 | AP002449.1 | 1.057906738 | 0.04095754 |
| ENSG00000260386 | LINC01225 | 1.050620613 | 0.03571136 |
| ENSG00000182326 | C1S | 1.002637354 | 0.02892392 |
| ENSG00000067955 | CBFB | 0.91210834 | 0.02818953 |
| ENSG00000185900 | POMK | 0.883451987 | 0.03992985 |
| ENSG00000181038 | METTL23 | -0.845987062 | 0.04983818 |
| ENSG00000271856 | LINC01215 | -0.899555025 | 0.04673993 |
| ENSG00000158485 | CD1B | -0.900513319 | 0.03881005 |
| ENSG00000152475 | ZNF837 | -0.904111373 | 0.0374028 |
| ENSG00000174165 | ZDHHC24 | -0.927486178 | 0.0279634 |
| ENSG00000250400 | LINC00977 | -1.008073285 | 0.04134606 |
| ENSG00000172456 | FGGY | -1.106808915 | 0.03029574 |
| ENSG00000229124 | VIM-AS1 | -1.107766272 | 0.03991628 |
| ENSG00000245954 | LINC02273 | -1.186171726 | 0.01161589 |
| ENSG00000254682 | AP002387.1 | -1.210785936 | 0.00531109 |
| ENSG00000138134 | STAMBPL1 | -1.22046974 | 0.00413692 |
| ENSG00000235423 | AC068768.1 | -1.223733801 | 0.01895017 |
| ENSG00000255857 | PXN-AS1 | -1.226015412 | 0.01900998 |
| ENSG00000276791 | AC092117.1 | -1.269703069 | 0.02185337 |
| ENSG00000280194 | AD000864.1 | -1.295049268 | 0.02586449 |
| ENSG00000225721 | AL592166.1 | -1.399319999 | 0.02668238 |
| ENSG00000143167 | GPA33 | -1.554908706 | 0.01230283 |
| ENSG00000161547 | SRSF2 | -1.592545626 | 0.0001586 |
| ENSG00000270332 | SMC2-AS1 | -2.049911172 | 0.04399131 |
| ENSG00000273154 | AL121845.3 | -2.049911172 | 0.04399131 |
| ENSG00000263884 | AP000845.1 | -2.192382366 | 0.01180303 |
| ENSG00000279494 | AL117328.2 | -2.466364115 | 0.00314942 |
| ENSG00000236269 | ENO1-IT1 | -2.903465354 | 0.00524731 |
| ENSG00000007062 | PROM1 | -3.000212227 | 0.0348666 |
| ENSG00000001626 | CFTR | -3.150223468 | 0.02156655 |
| ENSG00000230648 | AL138831.1 | -3.286095246 | 0.01341209 |
| ENSG00000242154 | AC004884.2 | -5.822613317 | 0.02267682 |
| ENSG00000260360 | AL353708.1 | -5.822613317 | 0.02267682 |

**Supplemental Materials and Methods**

**RNA-Seq**

Total RNA was extracted using RNAiso Plus (Takara, D9108B) according to the manufacturer’s instructions and RNA degradation and contamination were monitored on 1% agarose gels.

RNA purity was checked using the NanoPhotometer®spectrophotometer (IMPLEN, CA, USA).

The size and quality of RNA was assessed using the Agilent Bioanalyzer 2100 system (Agilent Technologies, CA, USA). Sequencing libraries were generated using NEBNext® UltraTM RNA Library Prep Kit for Illumina® (NEB, USA) following manufacturer’s protocol. Libraries were sequenced on the Illumina HiSeq 2500 platform. Differentially expressed genes were identified using the DESeq2 R package (1.16.1). Genes with P-adjust < 0.05 were considered as differentially expressed. The differentially expressed mRNAs were used for KEGG enrichment analysis by the clusterProfiler R package.

**ChIP-Seq**

ChIP assay was conducted according to Dahl’s protocol [56]. The chromatin DNA purity was checked using the NanoPhotometer® spectrophotometer (IMPLEN, CA, USA). DNA concentration was measured using Qubit® DNA Assay Kit in Qubit® 2.0 Flurometer (Life Technologies, CA, USA). Libraries were generated using Illumina’s TruSeq Library Prep Kit following instructions. Library quality was assessed on the Agilent Bioanalyzer 2100 system. Libraries were sequencd on the Illumina HiSeq platform at 150 bp pair-ended reads were generated. ChIP-Seq reads were quality controlled by FastQC performs basic statistics and Trimmomatic software. At last, Gene Ontology (GO) enrichment analysis was implemented by the GOseq R package, in which gene length bias was corrected. GO terms with corrected P-value less than 0.05 were considered significantly enriched by peak related genes.
